# Cancer‐Testis Antigens in Immuno Oncology Mechanisms Therapeutic Strategies and Clinical Perspectives

**DOI:** 10.1155/jimr/8131644

**Published:** 2026-09-17

**Authors:** Ranran Shi, Ling Ran, Tiantian Song, Xiaowen Zhou, Wenshan Zhao, Yijie Zhang

**Affiliations:** ^1^ Department of Basic Medical Sciences, Luohe Medical College, Luohe 462000, China, lhmc.edu.cn; ^2^ Henan Province Engineering and Technology Research Center of Foods for Special Medical Purpose, Luohe Medical College, Luohe 462000, China, lhmc.edu.cn; ^3^ Respiratory Medicine Department, Luohe Central Hospital, Luohe 462000, China, lhzxyy.com.cn; ^4^ School of Life Sciences, Zhengzhou University, Zhengzhou 450001, China, zzu.edu.cn; ^5^ Translational Medicine Center, Huaihe Hospital of Henan University, Kaifeng 475000, China, henu.edu.cn

**Keywords:** antigen-specific vaccines, cancer-testis antigens (CTAs), immune checkpoint inhibitors, tumor heterogeneity, tumor immunotherapy

## Abstract

Cancer‐testis antigens (CTAs) represent a unique class of tumor‐associated antigens (TAAs) with highly restricted expression in immune‐privileged tissues and widespread aberrant activation in various malignancies. Their potent immunogenicity and tumor specificity make CTAs promising targets for cancer immunotherapy. This review provides a comprehensive overview of the molecular regulation, biological functions, and therapeutic applications of CTAs. We detail the genetic and epigenetic mechanisms that drive CTA expression in cancer, their roles in tumor progression, and their capacity to elicit robust CD8^+^ T cell responses. Recent clinical advances in peptide‐ and dendritic cell–based vaccines, T‐cell receptor (TCR)‐engineered adoptive cell therapies, and combination strategies with immune checkpoint inhibitors (ICIs) are highlighted, emphasizing NY‐ESO‐1 and MAGE family antigens as leading candidates. We further discuss the major challenges limiting clinical translation, emphasizing that despite promising immunogenicity, most CTA‐directed modalities remain in early clinical evaluation with heterogeneous response rates, intratumoral heterogeneity, antigen loss, and risks of off‐target toxicity. The review also explores emerging approaches—such as epigenetic modulators, multiantigen targeting, and CRISPR‐based gene editing—that aim to enhance the precision and efficacy of CTA‐based immunotherapies. Together, these insights underscore the strategic potential of CTAs in next‐generation immunotherapy and outline the future directions required to bring these promising agents from bench to bedside.

## 1. Introduction

Cancer remains a leading cause of morbidity and mortality worldwide, accounting for over 19 million new cases each year and representing a formidable challenge in clinical oncology [1–3]. Conventional treatment modalities—including chemotherapy, radiotherapy, and surgical resection—have provided significant therapeutic benefit but are often undermined by their nonspecific targeting, toxicity to normal tissues, and limited ability to prevent recurrence [4]. Tumor heterogeneity, resistance mechanisms, and the plasticity of cancer cells further complicate effective disease control, driving an urgent demand for more precise, targeted approaches [5]. In this context, immunotherapy has revolutionized the landscape of cancer treatment [6]. By harnessing the immune system’s intrinsic capacity to recognize and eliminate malignant cells, immunotherapies such as immune checkpoint inhibitors (ICIs), CAR T‐cell therapy, and cancer vaccines have demonstrated durable responses across diverse tumor types [7, 8]. Despite these breakthroughs, immune evasion, antigen loss variants, and tumor‐mediated immunosuppression continue to hinder the long‐term success of immunotherapy [9, 10]. Thus, the identification and validation of tumor‐specific antigens remain critical to improving the selectivity, efficacy, and safety of immune‐based interventions [11].

Among tumor‐associated antigens (TAAs), cancer‐testis antigens (CTAs) have emerged as highly promising targets for immunotherapy owing to their uniquely restricted expression patterns and potent immunogenicity [12, 13]. CTAs are normally expressed only in immune‐privileged germline tissues such as the testis and are aberrantly re‐expressed in a wide variety of malignancies, including melanoma, lung, bladder, and ovarian cancers. While this differential expression profile was historically thought to minimize autoimmune toxicity, clinical experience with high‐affinity therapeutics has shown that low‐level somatic expression and unintended cross‐reactivity remain major safety concerns that require rigorous preclinical screening [14]. Many CTAs, including NY‐ESO‐1 and members of the MAGE‐A family, are transcriptionally regulated through epigenetic mechanisms such as DNA hypomethylation and histone acetylation, which facilitate their reactivation in tumor cells. Several of these antigens are highly immunogenic and capable of eliciting robust CD8^+^ T‐cell responses, making them particularly suitable for vaccine development and adoptive T‐cell strategies. Clinical observations support this potential: spontaneous T‐cell responses to CTAs have been detected in cancer patients, and high frequencies of NY‐ESO‐1–specific CD8^+^ T cells have been correlated with improved survival outcomes in melanoma and sarcoma [15]. These data collectively underscore the relevance of CTAs not only as biomarkers of tumor identity but also as actionable immunological targets with translational significance [16].

This review aims to provide a comprehensive and critical analysis of cancer/testis (CT) antigens as strategic targets in cancer immunotherapy. We will begin by examining the molecular and immunological underpinnings of CT antigen expression, with a particular focus on their regulation, immunogenicity, and tumor specificity. Subsequent sections will explore the therapeutic landscape of CT antigen‐directed interventions, encompassing cancer vaccines, T‐cell receptor (TCR)‐engineered adoptive T‐cell therapy, and combination strategies with immune checkpoint blockade (ICB). We will further address the biological and clinical challenges that limit the efficacy of CTA‐based therapies, such as antigen heterogeneity, immune tolerance, and cross‐reactivity risks—that currently constrain the therapeutic success of CTA‐based interventions. Finally, we will discuss emerging technologies—including multiomics profiling, antigen prediction algorithms, and gene editing tools—that offer new opportunities to enhance the precision and personalization of CTA‐targeted immunotherapy. By integrating molecular insights with translational advances, this review seeks to define the current state and future direction of CT antigen research in oncology.

## 2. Biology and Classification of CTAs

### 2.1. Genetic and Epigenetic Regulation of CTA Expression

CT antigens are expressed in a highly regulated manner, primarily under the control of genetic and epigenetic mechanisms. These genes are typically restricted to the testis, but during tumorigenesis, they are aberrantly reactivated in a variety of cancer cells. The expression of CT antigens is regulated through critical epigenetic modifications, such as DNA methylation and histone modifications [17]. In normal somatic cells, DNA methylation silences these genes, preventing their expression. However, in cancer cells, DNA demethylation or altered histone modifications can lead to the re‐expression of CT antigens, contributing to tumor progression [18, 19]. Recent studies have shown that DNA methylation and histone modifications play pivotal roles in silencing and reactivating CT antigens during tumorigenesis [20]. This epigenetic reprogramming is critical for understanding how these antigens become visible to the immune system in cancer cells and provide potential therapeutic targets for reprogramming tumor cell epigenetics to enhance immunogenicity [21, 22]. Furthermore, the regulation of these genes is essential for understanding their function and role in cancer progression.

CTAs are highly regulated by epigenetic mechanisms, particularly DNA methylation, which keeps these genes silent in normal tissues. In tumors, DNA demethylation leads to the re‐expression of CT antigens, making them visible to immune cells [23]. Histone modifications, such as acetylation and methylation, also influence the expression of CT antigens. By modifying these epigenetic patterns, researchers are exploring ways to reactivate CT antigens in tumors to enhance immune recognition, which may help boost the effectiveness of immunotherapy.

### 2.2. Expression Patterns and Functional Roles of CTAs

A fundamental question in CTA biology is whether their re‐expression represents a passive consequence of global cancer‐associated epigenetic alterations or an active driver of malignant progression. Accumulating evidence reveals a dual biological nature of CTAs: while genome‐wide DNA hypomethylation and histone modifications act as the upstream permissive trigger that unlocks CTA gene expression in somatic tissues [24, 25], reactivated CTAs subsequently function as active oncogenic drivers. Rather than remaining inert passenger proteins, specific CTAs directly engage key oncogenic signaling pathways—including the suppression of p53‐mediated apoptosis (MAGE family) [26], stimulation of cell cycle machinery (CAGE, CEP55) [27, 28], and activation of MAPK/Wnt cascades that induce epithelial–mesenchymal transition and metastasis (SSX2, CT45, and SPAG9) [29, 30]. Consequently, CTAs serve both as molecular readouts of epigenetic instability and as functional drivers that actively shape aggressive tumor phenotypes.

CT antigens are characterized by variable splice variants and phenotypic heterogeneity within tumor cells. They serve as potential biomarkers in various cancers, including prostate, cervical, breast, colorectal, gastric, bladder, liver, and lung cancers. Therefore, CTAs are promising targets capable of triggering specific immune responses in a wide range of tumors [16]. The broad expression of CTAs across different tumor types and stages suggests that CTA‐related genes play essential roles in tumor development. Tumor cells under metabolic pressure may escape antitumor processes by reactivating genetic programs related to gametogenesis and embryogenesis, thereby acquiring tumor‐specific features like migration and invasiveness. Studies have shown that CTAs also possess other functions beyond being immunogenic targets.

For instance, CTAs such as SSX and RAGE [31] are upregulated in multipotent mesenchymal stem cells but are suppressed in differentiated progeny [21]. SSX expression affects the transition between epithelial layers and mesenchymal stem cells [32]. SSX2 knockdown significantly inhibits melanoma cell proliferation and has been shown to activate MAPK and Wnt signaling pathways [33]. CAGE enhances the expression of Cyclin D1 and Cyclin E, promoting cell cycle progression. Additionally, CAGE contributes to angiogenesis, potentially supporting tumor growth [34]. Oncogenic CTAs like CT45 [29, 35], MAGE‐4 [36], PIWIL2 [37], SSX [38], CAGE, and CT45A1 [29] activate mitogen‐activated protein kinase and CREB signaling, induce epithelial‐mesenchymal transition (EMT), and promote cancer progression and metastasis [39].

MAGE family members regulate tumor cell survival. Knockdown of MAGE‐A, MAGE‐B, and MAGE‐C in melanoma cells disrupts the p53‐KAP1 complex, enhances p53 activity, and promotes apoptosis [40]. MAGE‐A directly inhibits the interaction between p53 and chromatin, suppressing its transcriptional regulation, and recruits transcriptional repressors to downregulate p53 function [41]. CTA expression is higher in metastatic melanoma compared to primary melanoma and nevi [42], suggesting a direct role in tumor invasion. Some CTAs, including PCA4 [43], SCP‐1 [44], SPANX [45], and CEP55 [46], have been linked to metastasis in various cancers, particularly colorectal cancer liver metastasis. The overexpression of CEP55 has been observed in multiple cancers such as breast, colorectal, and lung cancers, where it plays a crucial role in cell proliferation and prognosis. In colorectal cancer, CEP55 affects T‐cell response sensitivity and promotes proliferation via interaction with Plk1 and AKT1 pathways [47–49]. Likewise, CT antigen SPAG9 is linked to early stages of colorectal cancer and may serve as a diagnostic biomarker. SPAG9 interacts with JNK and is involved in the MAPK signaling pathway [50]. Its expression in renal cell carcinoma, cervical cancer, breast cancer, thyroid cancer, CML, and colorectal cancer indicates its clinical significance [51]. SPAG9 expression is detected in 74% of colorectal cancer tissues, and its knockdown inhibits proliferation, migration, and invasion of cancer cells. In prostate cancer, SPAG9 regulates angiogenesis and influences cell migration and invasion through modulation of MMPs [52].

### 2.3. Classification of CTAs

CT antigens can be broadly classified into two main categories: X‐linked and non‐X‐linked CT antigens. The X‐linked CT antigens, such as the MAGE family, are among the most studied due to their frequent expression across multiple tumor types, including melanoma and lung cancer [53]. These antigens are central to the development of vaccine‐based therapies and T‐cell therapy approaches. The MAGE family includes multiple members (e.g., MAGE‐A and MAGE‐B), each with varying roles in cancer immunogenicity [54, 55]. On the other hand, non‐X‐linked CT antigens, such as SSX, SCP‐1, and SPANX, also show significant therapeutic potential. These antigens demonstrate diverse expression profiles across different cancers, making them promising targets for combination immunotherapies. The differentiation between X‐linked and non‐X‐linked CT antigens provides an opportunity for developing personalized immunotherapies, where specific antigen profiles can be targeted based on each patient’s tumor characteristics. Ongoing research aims to explore and discover new CT antigens and understand their roles in tumor biology to expand the range of therapeutic options (Table 1) [42].

**Table 1:** Classification of cancer‐testis antigens.

Classification	Gene name	Tumor types associated	Immunogenicity	Current therapeutic strategies
MAGE (melanoma antigen gene)	MAGEA1, MAGEA3, MAGEC1	Melanoma, lung cancer, ovarian cancer	High	Peptide vaccines, TCR engineering, adoptive cell therapy
NY‐ESO (New York esophageal squamous cell carcinoma 1)	NYESO1, NYESO2	Melanoma, breast cancer, sarcoma	High	Peptide vaccines, dendritic cell therapy, immune checkpoint inhibitors
SSX (synovial sarcoma X)	SSX1, SSX2, SSX4	Synovial sarcoma, melanoma	Moderate to high	Peptide vaccines, TCR therapy
BAGE (B‐lymphocyte antigen gene expression)	BAGE1	Melanoma, bladder cancer	Moderate	Peptide vaccines, dendritic cell therapy
HAGE (human antigen gene)	HAGE	Melanoma, lung cancer	Moderate	Peptide vaccines, TCR engineering
PRAME (preferentially expressed antigen in melanoma)	PRAME	Melanoma, neuroblastoma	High	Peptide vaccines, ACT
GAGE (gastric cancer antigen)	GAGE	Melanoma, lung cancer	Moderate	Peptide vaccines, dendritic cell therapy
T‐cell receptor gamma gene	TCRG, TCRD	T‐cell lymphomas	High	TCR engineering, ACT
Other CT antigens	AFP	Liver cancer	Low	Targeted therapies

## 3. CTAs as Targets for Immunotherapy

### 3.1. Immunogenic Properties of CTAs

CTAs are distinguished not only by their restricted expression to tumors and immune‐privileged germline tissues but also by their potent immunogenicity [11, 56]. This unique combination makes them exceptionally well‐suited as immunotherapeutic targets. In contrast to many self‐antigens aberrantly expressed in tumors, CTAs are generally absent from somatic tissues and therefore not subject to central tolerance [57]. As a result, they are perceived by the immune system as nonself, enabling the generation of robust cellular and humoral immune responses. CTAs offer a compelling balance of tumor specificity and immunogenicity when compared to other tumor antigen classes, including TAAs and neoantigens, which differ markedly in their expression patterns, immunogenic profiles, and translational feasibility (Table 2).

**Table 2:** Types and characteristics of tumor antigen.

Comparison dimension	Cancer‐testis antigens (CTAs)	Tumor‐associated antigens (TAAs)	Neoantigens
Normal tissue expression	Expressed only in immune‐privileged tissues such as testis and placenta	Low expression in normal tissues	Not expressed in normal tissues
Tumor tissue expression	Widely expressed in various tumor types	Upregulated in multiple tumor types	Personalized, specific to tumor mutations
Immunogenicity	High, recognized as ”foreign antigens”	Relatively low, may induce immune tolerance	Very high, as “true non‐self antigens”
Therapeutic applicability	Broad, applicable to multiple tumor types	Broad, but risk of affecting normal tissues	Limited, requires personalized approach
Safety	High, minimal expression in normal tissues, low side effects	Moderate, may affect normal tissues	High, very low off‐target risk
Development complexity	Moderate, conserved antigens with established target libraries	Low, many known molecular targets	High, requires individual sequencing and screening
Representative targets	NY‐ESO‐1, MAGE‐A3, etc.	HER2, CEA, AFP, PSA, etc.	Unique to each patient (personalized)

**Figure 1:**
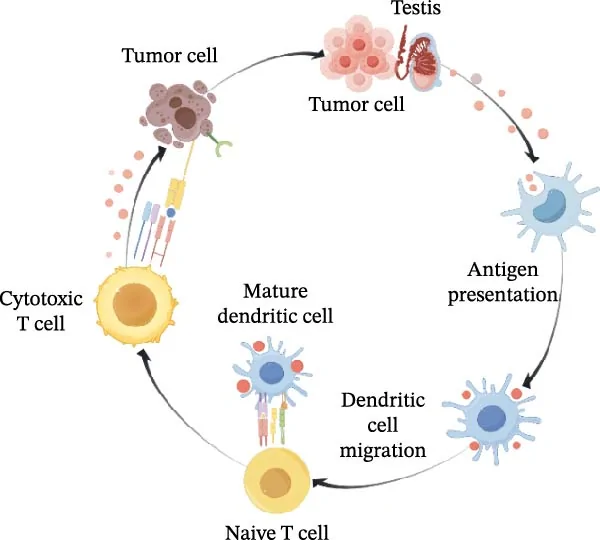
Mechanisms of cancer‐testis antigen presentation and T cell activation.

Mechanistically, CTAs are presented on the surface of tumor cells via major histocompatibility complex (MHC) class I molecules, facilitating recognition by cytotoxic CD8^+^ T cells [58]. In parallel, dendritic cells may cross‐present these antigens to prime naïve T cells and establish long‐term immunity—reflecting the coordinated antigen presentation pathways that underpins CTA‐specific T cell responses illustrated schematically in Figure 1.

For instance, the NY‐ESO‐1 antigen has been shown to induce specific T‐cell responses in patients with melanoma, leading to tumor regression and improved survival outcomes [59]. The immunogenicity of CT antigens not only allows for the development of cancer vaccines but also facilitates the design of adoptive T‐cell therapies. By engineering T cells to express receptors specific to CT antigens, researchers can enhance the specificity and efficacy of these therapies, providing a promising strategy for treating malignancies. Furthermore, the identification of epitopes derived from CT antigens, which are presented by MHC molecules on tumor cells, has opened new avenues for personalized immunotherapy approaches, wherein patients can receive tailored treatments based on their specific antigenic profiles [15, 60]. Recent advances show that MAGE‐A6 serves as a progenitor for SNP‐dependent neoepitope, proving that variant‐specific CTA epitopes can trigger potent immunogenic responses even in low tumor mutational burden (TMB) malignancies, thereby significantly broadening the applicability of precision CTA‐targeted therapies [61].

### 3.2. Clinical Progress in CTA‐Based Tumor Vaccines

A substantial body of preclinical and clinical evidence supports the immunogenicity of CTAs. CTAs are overexpressed in a variety of human cancers compared to adjacent nontumorous tissues, making them ideal targets for tumor vaccines [62, 63]. To better design immunotherapeutic strategies, the screening and identification of ideal tumor antigens are ongoing. While some CTAs are reported to have oncogenic roles, the function of many others remains unclear and warrants further investigation. Currently, CTA‐based vaccines have demonstrated strong immune responses and good tolerance in clinical trials. In a phase II trial involving metastatic colorectal cancer patients, long peptides derived from MAGE epitopes combined with OK432 and Montanide ISA‐51 showed promising clinical effects and low cytotoxicity [64].

NY‐ESO‐1 is among the most advanced CTAs in clinical development [59]. It is restricted in normal tissues but broadly expressed in tumors and elicits both humoral and cellular immune responses [65]. It is a promising candidate for tumor immunotherapy applicable to multiple tumor types [66]. Phase I trials of NY‐ESO‐1 revealed elevated serum levels in patients with lung, thyroid, ovarian, breast, bladder, esophageal cancers, and melanoma [15]. As of now, 107 open clinical trials on NY‐ESO‐1 are listed at clinicaltrials.gov (Table 3). HLA‐A2‐restricted peptides from NY‐ESO‐1/LAGE‐1 used in adoptive CD8^+^ T‐cell therapy prolonged survival in melanoma and synovial sarcoma patients [60].

**Table 3:** Overview of clinical trials targeting cancer‐testis antigens.

Trial name	CT antigen targeted	Phase	Indication	Number of participants	Intervention type	Outcome measures	Key findings
NCT01300273	NY‐ESO‐1	Phase 2	Advanced melanoma	50	Peptide vaccine (NY‐ESO‐1)	Immune response, Tumor response rate	60% of patients achieved significant immune response. Tumor regression observed in 30% of participants.
NCT01710259	MAGE‐A3	Phase 3	Nonsmall cell lung cancer	150	TCR therapy (MAGE‐A3 specific)	Safety, efficacy, overall survival	Promising response rate of 50%. Improved progression‐free survival compared to historical controls.
NCT02288867	SSX‐2	Phase 1	Synovial sarcoma	30	Dendritic cell vaccine (SSX‐2)	Safety, T‐cell activation	T‐cell responses observed in 75% of patients, indicating strong immunogenicity.
NCT02609386	PRAME	Phase 1/2	Metastatic melanoma	100	Combination of peptide vaccine + checkpoint inhibitor	Immune response, tumor response	Combination therapy showed improved response rates (65%) compared to peptide vaccine alone.
NCT02413624	GAGE	Phase 2	Bladder cancer	70	Peptide vaccine (GAGE)	Safety, efficacy, quality of life	40% of patients experienced tumor shrinkage; favorable safety profile.
NCT03267860	NY‐ESO‐1	Phase 1	Ovarian cancer	60	TCR therapy (NY‐ESO‐1 specific)	Safety, efficacy, overall survival	Significant tumor response observed in 45% of patients, with manageable side effects.
NCT02982805	HAGE	Phase 1	Melanoma	40	DNA vaccine (HAGE)	Safety, immune response	Induced immune responses in 70% of patients; ongoing monitoring for long‐term effects.
NCT02040223	BAGE	Phase 2	Non‐small‐cell lung cancer	80	Peptide vaccine (BAGE)	Safety, efficacy, progression‐free survival	Efficacy observed in 55% of patients with manageable toxicity.
NCT02164513	MAGE‐A3	Phase 1	Multiple myeloma	50	Dendritic cell vaccine (MAGE‐A3)	Safety, immune response	65% T‐cell activation noted; promising safety profile in this cohort.

Even patients unresponsive to ICIs may benefit from antigen‐specific immunotherapy [67]. For example, melanoma patients treated with CTLA‐4 monoclonal antibodies who also mounted a humoral response to NY‐ESO‐1 had better antitumor responses, and those with NY‐ESO‐1‐specific CD8^+^ T‐cell responses had longer survival [68]. Combining NY‐ESO‐1 vaccines with adjuvants and Th1‐enhancing agents prolonged survival in patients with skin, lung, ovarian, breast, and even bone or soft tissue sarcomas [69]. Vaccination with recombinant NY‐ESO‐1 in patients with advanced melanoma and ovarian cancer induced significant CD4^+^ and CD8^+^ T‐cell responses, with CD8^+^ responses correlating with progression‐free survival (PFS). Another strategy involved fusing NY‐ESO‐1 with a DEC‐205‐specific antibody along with a TLR agonist, which induced robust immune responses and disease stabilization in 13 of 42 patients [70].

### 3.3. Vaccine‐Based Immunotherapeutic Approaches Targeting CTAs

The strategic application of cancer‐testis (CT) antigens in vaccine‐based immunotherapy represents a major advancement in the development of tumor‐specific cancer treatments. Owing to their highly restricted expression in normal tissues and strong immunogenic potential, CT antigens such as NY‐ESO‐1 and MAGE‐A3 have emerged as leading candidates for cancer vaccine design [71]. These vaccines aim to stimulate a durable and antigen‐specific T‐cell response by priming the immune system to recognize tumor cells expressing these otherwise immunologically silent antigens [72]. Both preclinical and clinical investigations have shown promising results for CT antigen‐based vaccines. For instance, peptide vaccines incorporating immunodominant NY‐ESO‐1 epitopes have been reported to elicit potent CD8^+^ cytotoxic T‐cell and CD4^+^ helper T‐cell responses, particularly in patients with melanoma and synovial sarcoma, often correlating with improved progression‐free and overall survival [73, 74]. Similarly, dendritic cell vaccines pulsed with CT antigens or genetically engineered to express them have demonstrated enhanced antigen presentation capabilities and have been shown to induce robust T‐cell activation in clinical trials [75].

Despite these encouraging findings, significant challenges persist. Tumor heterogeneity, antigen loss variants, and immunosuppressive microenvironments continue to impede the long‐term efficacy of CT antigen‐targeted vaccines [76]. These limitations have led to the exploration of combinatorial strategies, including the integration of vaccines with ICIs [77], cytokine adjuvants [78], or epigenetic modulators [79], aimed at overcoming immune resistance and broadening therapeutic responses [80]. A wide array of therapeutic strategies has been developed to exploit CTAs as immunologic targets, encompassing platforms such as peptide‐based vaccines [81], DNA vaccines [82], dendritic cell therapies [83], adoptive cell transfer, and combination regimens with ICIs [84]. Each of these approaches engages distinct mechanisms of immune activation: peptide vaccines directly prime CD8^+^ T cells with defined epitopes [85]; DNA vaccines promote endogenous antigen expression; and dendritic cell–based platforms enhance antigen presentation to initiate robust adaptive responses. TCR‐engineered adoptive T‐cell therapies offer precision targeting of CTA‐expressing tumor cells, while checkpoint blockade may synergize with CTA‐specific immunity by amplifying T‐cell effector functions [86]. These modalities differ in their degree of personalization, immunogenic potential, production complexity, and risk of toxicity. Taken together, the therapeutic landscape for CTA‐based interventions reflects a balance between feasibility, immune activation strength, and clinical responsiveness across diverse cancer types and patient profiles (Table 4). In addition to the well‐characterized CTAs such as NY‐ESO‐1 and MAGE‐A3, several novel CTAs have demonstrated clinical promise as targets for peptide‐based vaccine strategies across a range of solid tumors. Among these, the DEPDC1‐derived peptide 294–302 (EYYELFVNI), restricted to HLA‐A
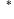
24, led to the development of the vaccine S‐288310, which showed favorable safety and prolonged survival in a phase I/II trial in patients with advanced bladder cancer [87]. Other CTAs—including LY6K, TTK, IMP3, and CDCA1—have also yielded evidence of tumor control and antigen‐specific immune responses in clinical studies involving head and neck, bladder, and other solid tumors [88]. In pancreatic cancer, a notoriously treatment‐refractory malignancy, a multipeptide vaccine targeting CDCA1, KIF20A, VEGFR2, and VEGFR1 demonstrated clinical benefit in a phase I trial [89]. Furthermore, acrosome binding protein (ACRBP) has recently been identified as a novel CTA candidate in ovarian cancer; its HLA‐A
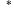
02:01‐restricted peptide elicits potent cytotoxic T‐cell responses and directly correlates with enhanced tumor immune cell infiltration [90]. These findings highlight the expanding immunotherapeutic potential of CTA‐derived peptides, and ongoing epitope discovery efforts continue to identify additional HLA‐restricted antigens with translational relevance [57].

**Table 4:** Comparison of therapeutic approaches involving cancer‐testis antigens.

Therapeutic approach	Description	CT antigens targeted	Mechanism of action	Clinical evidence	Advantages	Limitations
Peptide‐based vaccines	Vaccines composed of synthetic peptides derived from CT antigens that stimulate an immune response.	NY‐ESO‐1, MAGE‐A3, SSX	Induces CD8^+^ T‐cell activation against tumor cells expressing targeted CT antigens.	NY‐ESO‐1 vaccine trials show significant immune response and tumor regression in melanoma patients.	Minimal off‐target effects, targeted approach, potential for durable responses.	Variability in individual immune responses, risk of tolerance development, may require adjuvants.
DNA‐based vaccines	Vaccines that deliver plasmid DNA encoding CT antigens to induce immune responses.	MAGE‐A3, BAGE	Promotes the expression of CT antigens in host cells, facilitating T‐cell recognition.	Early‐phase trials indicate safety and immunogenicity in solid tumors.	Potential for long‐lasting immune memory, ease of production.	Limited initial immune response compared to peptide vaccines, challenges in delivery efficiency.
Dendritic cell‐based therapies	Autologous dendritic cells are loaded with CT antigens or their peptides to activate T‐cells.	NY‐ESO‐1, MAGE‐A3	Enhances the presentation of CT antigens to T‐cells, improving activation and expansion.	Clinical trials demonstrate strong T‐cell responses and some tumor regression.	Personalized treatment, strong activation of the immune system.	Labor‐intensive, expensive, and complex preparation process.
Adoptive cell therapy (ACT)	T cells engineered to express T‐cell receptors (TCRs) specific for CT antigens.	MAGE‐A3, NY‐ESO‐1	Directly targets tumor cells expressing specific CT antigens for destruction.	Trials show promising response rates in patients with metastatic melanoma.	High specificity and potency, potential for durable responses.	Complexity of TCR engineering, risk of off‐target effects and cytokine release syndrome.
Immune checkpoint inhibitors	Monoclonal antibodies that block inhibitory pathways (e.g., PD‐1/PD‐L1) to enhance T‐cell activity.	NY‐ESO‐1 (when coexpressed)	Releases the “brakes”on T‐cells, enhancing their ability to recognize and kill tumor cells.	Combination of anti‐PD‐1 with NY‐ESO‐1 vaccine shows improved outcomes in melanoma.	Broad applicability across various cancers, synergy with CT antigen‐targeted therapies.	Risk of autoimmune reactions, variability in patient responses.
Combination therapies	Strategies that integrate CT antigen‐targeted approaches with other therapeutic modalities.	NY‐ESO‐1, MAGE‐A3	Harnesses multiple mechanisms to enhance antitumor efficacy.	Ongoing trials exploring combinations with chemotherapy and checkpoint inhibitors.	Potential for synergistic effects, addressing tumor heterogeneity.	Increased complexity in treatment regimens, potential for cumulative toxicity.

**Figure 2:**
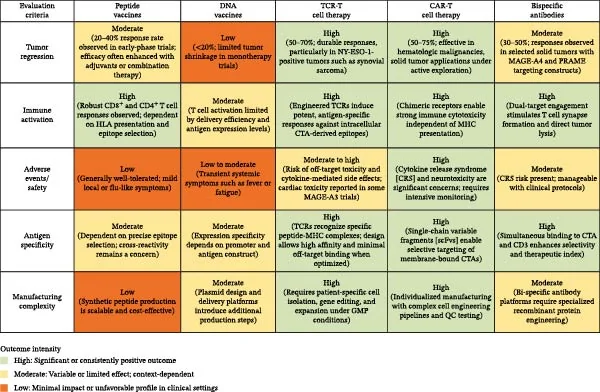
Comparative evaluation matrix of therapeutic strategies targeting cancer‐testis antigens.

The therapeutic landscape of CTA‐targeted strategies is diverse not only in platform design but also in key clinical performance metrics—including tumor regression, immune activation, safety profile, antigen specificity, and manufacturing complexity—across a range of cancer types and treatment modalities. This multidimensional perspective provides a basis for strategic comparison and prioritization of immunotherapeutic approaches (Figure 2) [91].

In addition to monotherapies centered on CT antigen‐targeted vaccines or engineered T cells, combination immunotherapy strategies have gained traction as a means to amplify antitumor immunity and overcome resistance mechanisms. Epigenetic modulators such as DNA methyltransferase inhibitors (DNMTi) can reverse gene silencing and restore the expression of CTAs, including MAGE, GAGE, NY‐ESO‐1, and PRAME. Upregulation of these CTAs by DNMT inhibitors has been associated with the induction of antigen‐specific T‐cell responses, especially in hematologic malignancies. However, caution is required as certain CTAs may also promote tumor progression by enhancing epithelial–mesenchymal transition (EMT) or metastasis. Therefore, the functional role of each CTA should be carefully studied before combining such agents with immunotherapy [80].

Another promising strategy is to integrate CTA‐based vaccines with ICIs. By increasing T‐cell infiltration and antigen presentation, CTA vaccines may enhance the responsiveness of tumors to PD‐1/PD‐L1 or CTLA‐4 blockade [67]. Clinical trials investigating such combinations are currently ongoing, particularly in tumors resistant to ICIs alone [92]. In addition, CTA‐based vaccines may enhance tumor‐infiltrating T‐cell responses and upregulate checkpoint molecule expression, thus potentially improving the efficacy of checkpoint blockade therapies. Rational sequencing of chemotherapy and CTA vaccination may help overcome immune resistance. Chemotherapy can selectively enrich for tumor clones expressing specific CTAs, making them more vulnerable to targeted T‐cell responses.

### 3.4. Adoptive Cell Therapy (ACT) Targeting CTAs

ACT represents a transformative approach in cancer treatment, and the incorporation of CT antigens as targets in this modality has shown considerable promise [93, 94]. ACT involves the isolation and expansion of immune cells, typically T cells, that are engineered or selected to specifically recognize and attack cancer cells expressing certain antigens. By targeting CT antigens, ACT leverages the unique immunogenicity of these tumor‐specific markers, leading to potent antitumor responses. One of the most notable advancements in this area has been the development of TCR engineered T cells, which are designed to recognize specific CT antigens presented by tumor cells.

Clinical trials involving TCR‐engineered T cells targeting CT antigens, such as NY‐ESO‐1 and MAGE‐A3, have demonstrated encouraging results, with patients experiencing significant tumor regressions and prolonged survival. In cutaneous melanoma, TCR‐T targeting NY‐ESO‐1 achieved a 28% objective response rate (ORR) and improved PFS [95]. MAGE‐A4‐targeted TCR‐T showed promise in synovial sarcoma (40%–60% ORR) and was FDA‐approved in 2024 [55].

The ability of these engineered T cells to selectively target tumor cells while sparing normal tissues is a critical advantage, reducing the risk of adverse effects often associated with traditional therapies. Moreover, recent innovations in CAR T‐cell therapy, which utilize chimeric antigen receptors to enhance T‐cell specificity and potency, have also begun to incorporate CT antigens, offering a promising avenue for more effective and personalized cancer treatments. However, challenges such as T‐cell persistence, potential off‐target effects, and tumor antigen heterogeneity remain areas of active research. Ongoing studies aim to optimize ACT protocols and explore combination strategies with other immunotherapies to maximize therapeutic benefits for patients.

### 3.5. The Role of CTAs in Combination With ICIs

The integration of cancer‐testis (CT) antigen targeting with ICB has emerged as a promising strategy to enhance the efficacy of cancer immunotherapy [96]. ICIs, particularly those targeting PD‐1/PD‐L1 and CTLA‐4 pathways, have demonstrated remarkable success in restoring antitumor T‐cell activity by counteracting tumor‐induced immune suppression. However, not all patients benefit from ICI monotherapy due to factors such as limited tumor immunogenicity and immune escape mechanisms [67, 97]. In this context, CT antigens offer a valuable complementary target due to their restricted expression in tumors and strong immunogenicity. Several preclinical and early‐phase clinical studies suggest that the presence of CT antigens may sensitize tumors to checkpoint blockade. CT antigens can enhance the visibility of tumor cells to the immune system, thereby increasing T‐cell infiltration and priming the immune microenvironment for a more effective ICI response. GDF1‐induced dedifferentiation in hepatocellular carcinoma (HCC) reactivates CTAs via the ALK7‐SMAD2/3 pathway, suppressing LSD1. This enhances tumor immunogenicity, creating a permissive environment for ICIs [98]. DUSP22‐rearranged lymphomas overexpress CTAs due to global DNA hypomethylation, concurrently downregulating PD‐L1 and upregulating costimulatory molecules. This suggests demethylating agents could augment ICI efficacy [99]. Liu et al. demonstrated that combining neoantigen vaccines with ICB synergistically enhanced antitumor immunity in preclinical models. The dual treatment promoted the expansion of a distinct subset of neoantigen‐specific CD8^+^ T cells exhibiting high cytotoxic potential (Fasl^+^, Gzma^+^), strong chemokine signaling (Cxcr3^+^, Ccl5^+^), and reduced expression of exhaustion markers (Lag3^−^, Havcr2^−^). This synergy was shown to depend on the effective priming of T cells in the tumor‐draining lymph nodes, providing mechanistic insight into how personalized cancer vaccines can be potentiated by concurrent ICB therapy [100]. This synergy not only potentiates antitumor immunity but also provides a rationale for combinatorial regimens tailored to immunogenic tumor subtypes [101]. Future research should aim to further define the immunological mechanisms underlying this synergy, identify predictive biomarkers such as CT antigen expression profiles, and evaluate optimal sequencing and dosing strategies for combination therapies. As the field advances, integrating CT antigen‐targeted approaches with ICIs holds the potential to expand the therapeutic benefit to a broader patient population, especially those with immune‐cold or refractory tumors [102].

## 4. Challenges and Limitations in Targeting CTAs

### 4.1. Tumor Heterogeneity and Antigen Escape

A major obstacle in CTA‐based immunotherapy is intratumoral heterogeneity and the associated risk of antigen escape. Tumors are composed of diverse cellular populations, and CTA expression can vary markedly among individual cancer cells. This heterogeneity undermines therapeutic efficacy as immune responses directed at a single CTA may spare antigen‐negative tumor subclones, allowing disease persistence and relapse [103].

Moreover, tumor cells can downregulate CTA expression, lose MHC class I molecules, or acquire mutations that modify immunogenic epitopes—mechanisms collectively facilitating immune evasion. Elucidating the genetic and epigenetic factors governing CTA expression is essential to developing robust, multiantigen targeting strategies that minimize escape and broaden immune coverage.

### 4.2. Immunosuppressive Tumor Microenvironment

The tumor immune microenvironment plays a critical role in the success of CTA‐based peptide vaccines. Therapeutic vaccines may perform better in adjuvant or minimal residual disease settings. In tumors with large burdens, combined therapeutic strategies are often necessary to debulk tumors and enhance immunogenicity [104, 105]. For instance, conventional chemotherapeutic and radiotherapeutic approaches remain effective for most colorectal cancer patients. However, for advanced or recurrent cases, resistance to chemotherapy is a major challenge. Immunotherapy in combination with chemotherapy may eliminate residual tumor cells and improve patient survival. Since chemotherapeutic agents induce apoptosis through a variety of mechanisms, the selection of antiapoptotic CTAs as immunotherapy targets may synergize with chemotherapy by targeting residual tumor cells that escape cytotoxic agents [106].

### 4.3. Autoimmunity and Off‐Target Effects

While CT antigens offer a promising avenue for targeted cancer immunotherapy, the potential for autoimmunity and off‐target effects remains a critical concern. The expression of CT antigens is primarily restricted to immune‐privileged sites such as the testis; however, the immune system may inadvertently recognize these antigens as targets, leading to autoimmune reactions [107]. Autoimmunity can manifest as various adverse effects, ranging from mild to severe, depending on the extent of the immune response and the tissues involved [108]. For instance, patients undergoing immunotherapy targeting CT antigens may develop autoimmune disorders affecting the skin, endocrine glands, or other organs, complicating the treatment landscape and necessitating careful patient monitoring.

To mitigate the risk of autoimmunity, it is essential to carefully evaluate the safety profiles of CT antigen‐targeted therapies during clinical trials. Strategies such as identifying patient‐specific immune responses and developing more selective targeting approaches can help minimize the likelihood of off‐target effects. Additionally, ongoing research into the immunogenicity of CT antigens may lead to the identification of specific epitopes that are less likely to induce autoimmune reactions, providing a pathway for safer and more effective therapies. As the field of cancer immunotherapy continues to advance, addressing the challenges of autoimmunity will be critical to ensuring the success of CT antigen‐targeted approaches in clinical practice [109].

### 4.4. On‐Target, Off‐Tumor Toxicity and Safety Challenges

While antigen loss and the immunosuppressive tumor microenvironment pose major barriers to therapeutic efficacy, on‐target, off‐tumor toxicity, and off‐target cross‐reactivity represent paramount safety concerns in the clinical translation of CTA‐directed immunotherapies. Although CTAs are historically characterized by their restricted expression in immune‐privileged germline tissues, low‐level or heterogeneous expression can occasionally occur in vital somatic tissues. When using high‐affinity therapeutic modalities—such as engineered T‐cell receptor (TCR)‐T cells or bispecific antibodies—even trace amounts of CTA expression in normal tissues can trigger severe, life‐threatening autoreactive toxicities.

Notable clinical examples of these safety challenges were prominently demonstrated in early clinical trials targeting MAGE‐A3. In a trial evaluating an affinity‐enhanced HLA‐A
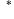
01‐restricted MAGE‐A3 TCR, patients experienced fatal cardiogenic shock and severe myocarditis. Subsequent mechanistic investigations revealed that TCR affinity maturation had induced unexpected off‐target cross‐reactivity against a nonidentical peptide derived from Titin (ESDPIVAQY)—a critical structural protein expressed in human cardiomyocytes—which had gone undetected during initial preclinical alanine‐scanning assays [110, 111].

Similarly, severe neurotoxicity was reported in another clinical study utilizing an HLA‐A
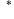
02:01‐restricted TCR designed to target MAGE‐A3, MAGE‐A9, and MAGE‐A12. In this trial, several patients developed severe neurological complications and subsequently died from necrotizing leukoencephalopathy. This fatal outcome was driven by the unrecognized, low‐level expression of MAGE‐A12 in normal human brain tissue, which triggered autoreactive T‐cell infiltration and central nervous system damage [112].

These cases underscore that high‐affinity TCR engineering can inadvertently broaden cross‐reactivity profiles and recognize shared epitopes in somatic tissues. Consequently, ensuring stringent tumor specificity, conducting comprehensive human tissue cross‐reactivity profiling, and implementing rigorous preclinical safety screening are crucial in advancing CTA‐based therapeutics.

### 4.5. Mechanisms of Immune Evasion in Tumors

Tumors have evolved various mechanisms to evade immune surveillance, posing significant challenges for the successful targeting of CT antigens in immunotherapy [113, 114]. These immune evasion strategies can significantly dampen the effectiveness of immune‐based treatments and are critical factors to consider in the design of therapeutic interventions [115]. One prominent mechanism is the upregulation of immune checkpoint molecules, such as PD‐L1 and CTLA‐4, which inhibit T‐cell activation and function. Tumors expressing CT antigens may exploit this pathway to suppress the immune response, allowing them to escape detection and destruction by the immune system.

**Figure 3:**
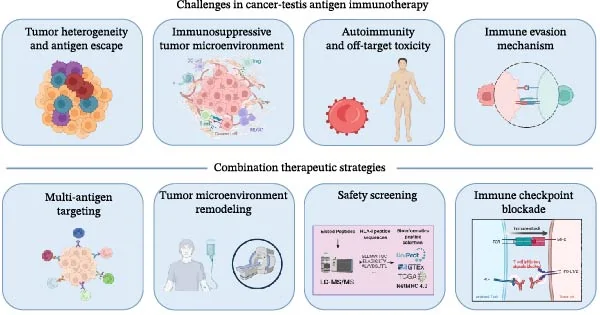
Challenges and combination strategies in cancer‐testis antigen (CTA)‐based immunotherapy.

Furthermore, tumor microenvironments are often characterized by immunosuppressive factors, including regulatory T cells (Tregs) and myeloid‐derived suppressor cells (MDSCs), which further inhibit effective antitumor immunity [116, 117]. These cells can secrete cytokines that suppress T‐cell activation or promote an environment conducive to tumor growth [118]. Understanding these immune evasion mechanisms is vital for developing effective therapeutic strategies that can overcome these barriers. Combining CT antigen‐targeted therapies with ICIs or agents that modulate the tumor microenvironment may enhance the overall effectiveness of treatment by reinvigorating the immune response. Ongoing research in this area aims to elucidate the complex interactions between tumors and the immune system, paving the way for innovative combination therapies that can enhance patient outcomes. These approaches are increasingly being shaped by an integrated view of immunological bottlenecks, therapeutic targets, and synergistic treatment modalities that define the current direction of CTA‐based immunotherapy (Figure 3).

## 5. Conclusion

CTAs have emerged as a compelling class of TAAs due to their restricted expression in immune‐privileged germline tissues and aberrant activation in a broad range of malignancies [119, 120]. Their lack of expression in most normal somatic tissues provides an immunological window that reduces the risk of autoimmunity while preserving high immunogenicity. This unique profile has enabled the development of a diverse array of CTA‐targeted immunotherapeutic strategies [121]. Over the past two decades, these include peptide‐ and DNA‐based vaccines, dendritic cell therapies, and engineered T‐cell modalities such as TCR‐T and CAR‐T therapies. Notably, NY‐ESO‐1 and MAGE‐A3 have served as model antigens, demonstrating consistent immune responsiveness and clinical activity in early‐phase trials across melanoma, sarcoma, and non‐small‐cell lung cancers [35]. The broad interest in CTAs reflects their versatility: they function both as shared tumor targets for off‐the‐shelf therapies and as candidates for personalized antigen design.

The therapeutic promise of CTAs is further enriched by advances in epigenetic modulation and combination immunotherapy. Agents such as DNA methyltransferase inhibitors (DNMTi) have been shown to restore CTA expression in epigenetically silenced tumors, enhancing antigen presentation and T‐cell infiltration. Such reprogramming strategies are especially valuable in “cold” tumors with poor immunogenicity [122], such as certain subtypes of colorectal or pancreatic cancers [123]. Concurrently, the combination of CTA‐targeted therapies with ICIs (e.g., anti–PD‐1 and anti–CTLA‐4) offers a synergistic approach to overcome immune resistance. CTA‐directed vaccines may potentiate checkpoint blockade by expanding tumor‐specific T‐cell populations and modifying the tumor immune landscape [124]. These combination strategies are currently under active clinical investigation, with early results suggesting improved response rates even in patients who had previously failed monotherapies.

Emerging evidence also points to a growing repertoire of novel CTAs—such as DEPDC1 [125], TTK [126], and CDCA1 [127]—that exhibit immunogenic potential in diverse solid tumors. Phase I/II trials investigating multipeptide vaccines or TCR‐transduced T cells targeting these newer antigens have reported encouraging outcomes, including immune activation, tumor control, and improved survival in selected patient populations [128]. However, the biological complexity of CTAs demands careful consideration. Some members of the CTA family are implicated in tumor proliferation, epithelial–mesenchymal transition (EMT), or metastatic dissemination. These dual roles necessitate functional characterization prior to clinical deployment. Additionally, challenges such as antigen loss, tumor heterogeneity, MHC restriction [129], and manufacturing complexity remain major obstacles to widespread application. Addressing these issues will require not only technological innovation but also the development of robust biomarker platforms for patient selection and real‐time response monitoring.

**Figure 4:**
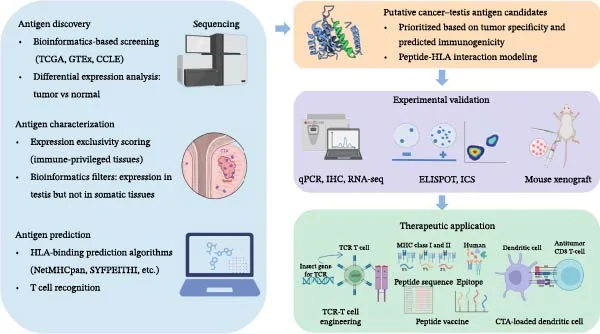
Comprehensive pipeline of CTA‐based immunotherapeutic development.

In summary, CTAs occupy a unique and strategic position in the evolving landscape of cancer immunotherapy [55, 130, 131]. They serve as both a molecular hallmark of malignant transformation and a practical therapeutic entry point for immune‐based interventions. With continued progress in antigen discovery, epigenetic reprogramming, and combinatorial immunotherapy, the full therapeutic potential of CTAs is becoming increasingly accessible [132]. The integration of CTAs into personalized oncology frameworks—supported by high‐throughput immunogenomic profiling, rational antigen selection, multiplatform validation, and adaptive clinical trial designs—has led to a progressively structured development path for CTA‐based therapies (Figure 4). As the field moves toward more precise and durable forms of tumor‐specific immunologic control, CTAs are poised not only to complement existing therapeutic paradigms but also to help define the next generation of antigen‐guided cancer immunotherapies.

## Author Contributions

Ranran Shi conceived the review topic, performed the literature search, drafted the manuscript, prepared the figures, and coordinated the writing process. Ling Ran contributed to data collation, literature analysis, and assisted in figure development and manuscript editing. Xiaowen Zhou participated in critical reading of the manuscript and contributed to section‐specific content refinement. Wenshan Zhao provided immunological expertise and guided the conceptual framework related to antigen processing and T‐cell responses. Yijie Zhang provided clinical insight into cancer immunotherapy, contributed to translational relevance, and reviewed the manuscript for clinical accuracy and coherence. All authors read and approved the final version of the manuscript.

## Funding

This work was supported by the National Natural Science Foundation of China (Grant 82404497), Doctoral Scientific Research Start‐up Foundation of Luohe Medical College (Grant 2022‐DF‐02), Science and Technology Research Project of Henan Provincial Department of Science and Technology (Grant 252102310393), Key Research Projects of Higher Education Institutions in Henan Province (Grants 24B310002 and 25A180007), Luohe Medical College Science and Technology Innovation Project (Grants 2023ZD01 and KJCX202401), Natural Science Foundation of Henan Province (Grant 252300420627).

## Consent

All authors consent to the submission of the article for publication.

## Conflicts of Interest

The authors declare no conflicts of interest.

## Data Availability

The data that support the findings of this study are available from the corresponding author upon reasonable request.
